# Supplementary material for: Causal Relationship between Aging and Anorexia Nervosa: A White-Matter-Microstructure-Mediated Mendelian Randomization Analysis
Source: Biomedicines. 2024 Aug 16;12(8):1874. doi: 10.3390/biomedicines12081874 (PMC11351342; doi:10.3390/biomedicines12081874)
Supplement: Supplementary file 1 [file biomedicines-12-01874-s001.zip › Supplementary Figure/Figure S4/Singe SNP plot of MR analysis for TL to ubm-b-1479.pdf]

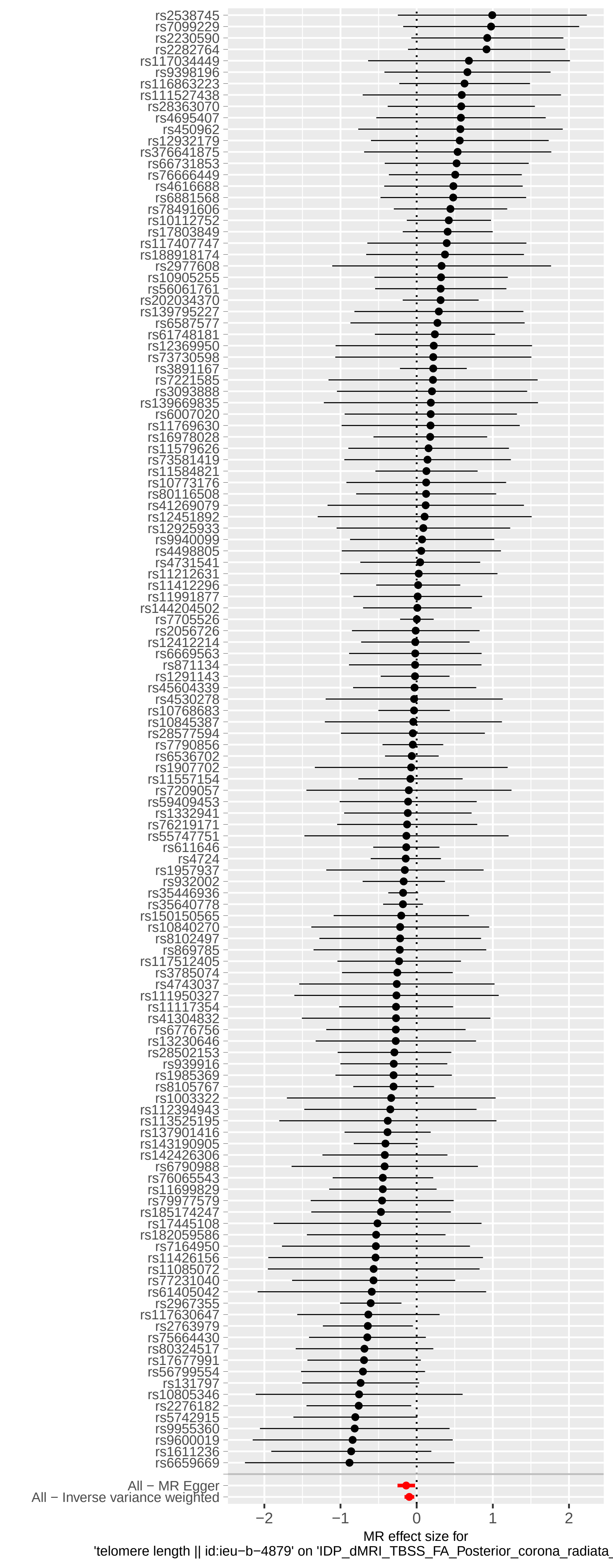

rs2538745
rs7099229
rs2230590
rs2282764
rs117034449
rs9398196
rs116863223
rs111527438
rs28363070
rs4695407
rs450962
rs12932179
rs376641875
rs66731853
rs76666449
rs4616688
rs6881568
rs78491606
rs10112752
rs17803849
rs117407747
rs188918174
rs2977608
rs10905255
rs56061761
rs202034370
rs139795227
rs6587577
rs61748181
rs12369950
rs73730598
rs3891167
rs7221585
rs3093888
rs139669835
rs6007020
rs11769630
rs16978028
rs11579626
rs73581419
rs11584821
rs10773176
rs80116508
rs41269079
rs12451892
rs12925933
rs9940099
rs4498805
rs4731541
rs11212631
rs11412296
rs11991877
rs144204502
rs7705526
rs2056726
rs12412214
rs6669563
rs871134
rs1291143
rs45604339
rs4530278
rs10768683
rs10845387
rs28577594
rs7790856
rs6536702
rs1907702
rs11557154
rs7209057
rs59409453
rs1332941
rs76219171
rs55747751
rs611646
rs4724
rs1957937
rs932002
rs35446936
rs35640778
rs150150565
rs10840270
rs8102497
rs869785
rs117512405
rs3785074
rs4743037
rs111950327
rs11117354
rs41304832
rs6776756
rs13230646
rs28502153
rs939916
rs1985369
rs8105767
rs1003322
rs112394943
rs113525195
rs137901416
rs143190905
rs142426306
rs6790988
rs76065543
rs11699829
rs79977579
rs185174247
rs17445108
rs182059586
rs7164950
rs11426156
rs11085072
rs77231040
rs61405042
rs2967355
rs117630647
rs2763979
rs75664430
rs80324517
rs17677991
rs56799554
rs131797
rs10805346
rs2276182
rs5742915
rs9955360
rs9600019
rs1611236
rs6659669
All − MR Egger
All − Inverse variance weighted
−2
−1
0
1
2
MR effect size for
'telomere length || id:ieu−b−4879' on 'IDP_dMRI_TBSS_FA_Posterior_corona_radiata_
